# Reservoir Host Expansion of Hantavirus, China

**DOI:** 10.3201/eid2101.140960

**Published:** 2015-01

**Authors:** Li-Zhu Fang, Li Zhao, Hong-Ling Wen, Zhen-Tang Zhang, Jian-Wei Liu, Shu-Ting He, Zai-Feng Xue, Dong-Qiang Ma, Xiao-Shuang Zhang, Yan Zhang, Xue-jie Yu

**Affiliations:** School of Public Health, Shandong University, Jinan, China (L.-Z. Fang, L. Zhao, H.-L. Wen, J.-W.. Liu, S.-T. He, X.-S. Zhang, X.-j. Yu);; Huangdao District Center for Disease Control and Prevention, Qingdao City, China (Z.-T. Zhang, Z.-F. Xue, D.-Q. Ma);; College of Medicine and Nursing, Dezhou University, Dezhou City, China (Y. Zhang);; University of Texas Medical Branch, Galveston, Texas, USA (X.-j. Yu)

**Keywords:** Hantavirus, seroprevalence, shrew, rodent, China, viruses

**To the Editor:** Hemorrhagic fever with renal syndrome (HFRS) is caused by hantavirus. During 1995–2005, China reported 20,000–50,000 cases of HFRS annually, which represents 90% of HFRS cases worldwide (*1*–*3*). In China, HFRS is caused mainly by 2 serotypes of hantavirus: Hantaan virus (HTNV) and Seoul virus (SEOV) (*4*). Pathogenic hantavirus serotypes are considered to be strictly associated with their serotype-specific reservoir hosts. HTNV is associated with the striped field mouse (*Apodemus agrarius*), and SEOV is associated with the brown rat (*Rattus norvegicus*) and the black rat (*Rattus rattus*) (*4*,*5*). HTNV causes a severe form of HFRS, characterized by renal failure that in some cases is followed by pulmonary edema and disseminated intravascular coagulation; the estimated death rate is 5%–15%. SEOV causes a moderate form of HFRS (*6*).

Jiaonan County in Shandong Province is one of the high-incidence HFRS areas in China. To detect the hantavirus infection in small mammals, we trapped rodents and shrews during December 2012–November 2013 using snap-traps in Jiaonan County (longitude 119°30′–120°30′, latitude 35°35′–36°08′).

We captured 1,276 animals comprising 5 rodent species and 1 shrew species (Table) and analyzed serum antibody against hantavirus of each animal using an antigen sandwich ELISA Kit (Shanghai Jiahe Biotechnology, Shanghai, China). The serum was considered to contain antibodies against hantavirus when the optical density (OD)_450nm_ of the sample was greater than the threshold. The threshold was calculated by using the equation: threshold = the average OD of the negative control + 0.15. ELISA results showed that 23.3% of animals were seropositive to hantavirus antigen (Table). The seropositive rate to hantavirus was 44.0% in Asian house shrews (*Suncus murinus*), 25.3% in house mice (*Mus musculus*), 15.4% in Chinese hamsters (*Cricetulus griseus*), 10.3% in brown rats, 10.1% in striped field mice (*Apodemus agraius*), and 3.0% in greater long-tailed hamsters (*C. triton*). The seropositivity rate for rodents was higher during summer (May–August) and lower during spring (March and April) and winter (October and November) but not significantly different among the months.

**Table:** Seropositive rate and RT-PCR–positive rate of hantaviruses in small mammals, Jiaonan County, China, December 2012–November 2013

Animal species	No. (%) animals	Seroprevalence of hantavirus	No. tested/no. RT-PCR positive (%)	
			HTNV	SEOV
*Apodemus agrarius*	268 (21.0)	27 (10.1)	12/191 (6.3)	0/191
*Cricetulus griseus*	156 (12.2)	24 (15.4)	0/63	0/63
*C. triton*	135 (10.6)	4 (3.0)	0/48	0/48
*Mus musculus*	245 (19.2)	62 (25.3)	2/143 (1.4)	0/143
*Rattus norvegicus*	213 (16.7)	22 (10.3)	1/159 (0.6)	13/159 (8.2)
*Suncus murinus*	259 (20.3)	114 (44.0)	0/121	2/121 (1.7)
Total	1,276 (100)	253 (19.8)	15/725 (2.1)	15/725 (2.1)

*HTNV, Hantaan virus; RT-PCR, reverse transcription PCR; SEOV, Seoul virus.

To determine what types of hantavirus infected the animals, we amplified viral RNA of HTNV and SEOV from animal lung samples using reverse transcription PCR with serotype-specific primers (*7*); 2.1% of animals had viral RNA of HTNV, and 2.1% had viral RNA of SEOV (Table). HTNV RNA was detected in striped field mice (6.3%), house mice (1.4%), and brown rats (0.6%). The hantavirus-positive animals were captured in February, April, and November for stripped field mice; November for brown rats; and April and November for house mice. SEOV was detected in brown rats (8.2%) and Asian house shrews (1.7%). These SEOV-positive animals were captured in January, March, May, June, and July for brown rats and March and November for Asian house shrews. The phylogenetic analysis of sequences amplified by reverse transcription PCR is presented in the Technical Appendix Figure. The nucleotide sequences of the PCR products have been deposited in GenBank (accession nos. KM357423–KM357452).

Hantavirus had been considered to be strictly associated with specific reservoir hosts and to have the same geographic distribution pattern as these reservoir hosts. All hantaviruses that caused human diseases had been associated with rodents, including members of *Murinae*, *Arvicolinae*, and *Sigmodontinae* spp. Insectivore hantaviruses were not known to cause human disease. The rodent hantavirus and the insectivorous hantaviruses were thought to have co-evolved with their specific rodent and insectivorous hosts over millions of years (*8*). One observed geographic clustering of hantavirus strains, and the association of hantaviruses with their reservoirs, might have been caused by an isolation-by-distance mechanism (*9*,*10*) and mixture of both host switching and co-divergence (*10*). Our study demonstrated that HTNV not only infects its traditional host, the striped mouse, but also infects house mice and rats; SEOV infects not only rats but also shrews, suggesting host expansion for both HTNV and SEOV in China. Our hypothesis is that the hantaviruses co-evolved with their animal hosts, such as SEOV with rats and HTNV with striped mice, but when their animal hosts expanded their territory, hantavirus had more chance to infect other susceptible rodents and expanded their animal hosts.

Both Asian house shrews and house mice are closely associated with humans by living inside and outside of human houses in China. The Asian house shrew and house mouse have been underestimated as potential animal hosts of SEOV and HTNV. To our knowledge, only 1 previous study had associated Asian house shrews with SEOV; in that study, an SEOV strain was isolated from an Asian house shrew in China (*2*).

Technical Appendix Phylogenetic tree of hantaviruses from small mammals, Jiaonan County, China, December 2012–November 2013.
