# Supplementary material for: Reservoir Host Expansion of Hantavirus, China
Source: Emerg Infect Dis. 2015 Jan;21(1):170–1. doi: 10.3201/eid2101.140960 (PMC4285249; doi:10.3201/eid2101.140960)
Supplement: Technical Appendix — Phylogenetic tree of hantaviruses from small mammals, Jiaonan County, China, December 2012–November 2013. [file 14-0960-Techapp-s1.pdf]

Article DOI: http://dx.doi.org/10.3201/eid2101.140960

Reservoir Host Expansion of Hantavirus, China

Technical Appendix

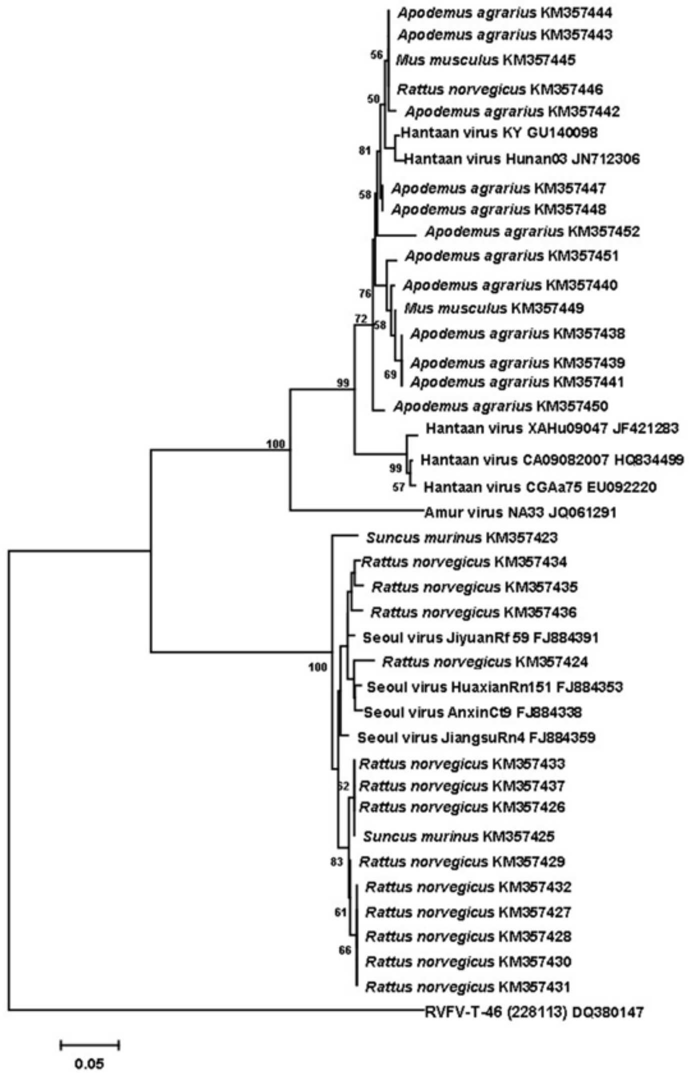

Technical Appendix Figure. Phylogenetic tree of hantaviruses from small mammals, Jiaonan County, China, December 2012–November 2013. A maximum-likelihood tree was constructed by using MEGA5 software (http://www.megasoftware.net) with 2,000 replicates for bootstrap testing. The tree was rooted with Rift Valley fever virus (GenBank accession no. DQ380147.1). Scale bar indicates nucleotide substitutions per site.
